# The gut–bone axis in osteoporosis: a multifaceted interaction with implications for bone health

**DOI:** 10.3389/fendo.2025.1569152

**Published:** 2025-07-16

**Authors:** Chunli Gu, Hong Du, Ningying Li, Yunlong Zhou, Sha Li, Yuchen Sun, Yiyang Han, Xuan Xu, Xianrong Li

**Affiliations:** ^1^ School of Nursing, Southwest Medical University, Luzhou, Sichuan, China; ^2^ Department of Orthopedics, The People’s Hospital of Leshan, Leshan, Sichuan, China; ^3^ School of Clinical Medicine, Southwest Medical University, Luzhou, Sichuan, China; ^4^ Department of Clinical Medicine, University of Hong Kong, Hongkong, Hongkong SAR, China; ^5^ Institute of Translational Medicine, Jiangxi Medical College, Nanchang University, Nanchang, Jiangxi, China

**Keywords:** gut-bone axis, osteoporosis, gut microbiota, immune and endocrine regulation, orthopedic diseases, bone metabolism, bone health

## Abstract

With the accelerated aging of the population, degenerative orthopedic diseases, particularly osteoporosis, have become a major public health challenge, threatening bone health and affecting the quality of life. Existing anti-osteoporosis regimens remain rather unitary or poorly adhered, which also limits the maintenance of bone health to some extent. Given the increasingly elucidated prominence of gut-related factors in osteoblasts/osteoclasts and bone formation/metabolism/maintenance, focusing on intestinal microecology and then targeting the distal bone tissue *via* the gut–bone axis have been recognized as a feasible intervention strategy. This review systematically summarized the interaction of the gut–bone axis while highlighting the physicochemical barriers formed by intestinal intrinsic structures, the gut microbiota, and related molecules for bone health maintenance through the immune and endocrine pathways. Meanwhile, we emphasized the ideal anti-osteoporotic property and individual achievability of methods like fecal microbiota transplantation, probiotic and prebiotic supplementation, and dietary pattern modification. The conceptual framework of the gut–bone axis plus X was innovatively proposed, given the potential synergy among different organs in disease characterization and pathogenesis, which may help better explain the etiology and manage other co-morbidities concurrent with or secondary to osteoporosis. Since the intersection of orthopedics with other subjects, we also supported the application of nano-biomaterials, bacterial synthetic biology, and novel small molecules in anti-osteoporosis, which is expected to unlock broader prospects for the multidisciplinary integration of the gut–bone axis.

## 1 Introduction

Bones are integral to multiple systems of the human body, such as locomotion, hematopoiesis, and metabolism (1), whose formation and maintenance affect life activities both structurally and functionally. Irrespective of the slow accumulation of tiny lesions or their instantaneous aggressive attacks, the qualitative alterations caused by quantitative changes in the human skeleton often, in turn, result in the occurrence and prolongation of osteoporosis, osteoarthritis, fractures, and other orthopedic diseases (2). Osteoporosis (3), a systemic degenerative bone disease characterized by declining bone mass and microstructural disruption, has become a major health concern, hindering the self-management of aging populations, with an incidence of more than 200 million people worldwide (4). Due to the fixed skeletal position and its dynamic pathophysiological behavior, the diagnosis and monitoring of musculoskeletal injuries generally rely on X-rays, CT, and MRI, while the treatment and prognosis involve uncertainty to some extent (5). Notably, the gut, as an organ distant from the skeleton, may possess a unique strength in extending the therapeutic spectrum of acute and chronic orthopedic diseases by functional linkages.

The human gut is recognized as a multi-constituent and multi-functional digestive organ (6). Inherent structures such as the intestinal epithelium, enteric nerves, gut microbiome, and derived metabolites, together with their interactions therein, constitute the gut microecology collectively (7). With the combination of omics and sequencing (8), the evolutionary patterns of the intestinal microenvironment in different somatic states have been clearly understood. Except intestinal pathologies like inflammatory bowel disease (IBD) and irritable bowel syndrome (IBS) (9, 10), varying degrees of intestinal dysbiosis also exist in other systemic diseases including obesity, diabetes, and Parkinson’s disease, which may be attributed to the metabolic, immune, and neural pathways (11). Hence, the concept of the gut-X axis is represented by the gut–brain axis (12), which refers to the dual communication between the gut and brain *via* a neuroendocrine pathway, externally impacting mood, cognition, and behavior, and vice versa. In orthopedics, postmenopausal osteoporosis (PMOP) is associated with a significant decline in the diversity and abundance of intestinal microbial communities in a clinical study enrolling 106 individuals, while bone loss was positively correlated with the decrease in genera *Allisonella*, *Klebsiella*, and *Megasphaera* (13). In contrast, Guan Z et al. (14) reviewed the effect of the gut microbiota depletion on osteoporosis and osteoarthritis, mentioning that symptoms such as reduced bone mineral density (BMD) were induced in a mouse model of post-antibiotic microbial depletion. The above hints at the plausibility of establishing an axial system between the gut and bone tissue, which may benefit interventions for osteoporosis.

This subtle link between intestinal ecology and the bone microenvironment is defined as the gut–bone axis (15), which has revolutionized the management principle of bone-related diseases. Microbes and their metabolites originating from the gut exhibit local or distal effects on the bone by repairing the integrity of the intestinal epithelial barrier, participating in neurological, immune, and endocrine regulation; restoring the homeostasis between osteogenesis and osteoblasts; and ultimately prompting the long-term maintenance of bone health (16). Several studies have reported that metabolic bone diseases such as osteoporosis and osteoarthritis would be alleviated or even reversed through strategies targeting the gut–bone axis, as evidenced by inhibited bone loss, fortified bone biomechanics, and increased trabecular numbers (17). A shortcoming, however, is that current literature on the relevance of the gut–bone axis in orthopedic diseases remains insufficient and lacks a more comprehensive synthesis on how to combine the two or how to combine them with other organs.

Therefore, this review focused on orthopedic diseases, particularly osteoporosis; emphasized the effects of intestinal factors (such as gut microbiota, derived metabolites, and gut barrier) on osteogenesis, bone metabolism, and maintenance *via* the gut–bone axis; and provided feasible insights into the classical or innovative interventions that target intestinal microecology to mitigate osteoporosis and restore bone health. In addition, we expanded knowledge of the gut–bone axis and systematically generalized the potential routes of the gut–bone axis plus X, which may benefit the co-treatments of co-morbidities secondary or concomitant to orthopedic diseases.

## 2 Perceptions on osteoporosis and gut–bone axis

To tackle osteoporosis, the first step is to know how to assess bone health in different stages of bone growth and define what parameters to adopt. Considering both histological structure and physiological function, a healthy skeleton indicates the compositional integrity and excellent adaptation of the bone (18), that is, having an ideal height and shape and being moderately tough, which requires the bone to have sufficient density and strength to support the body, protect the internal organs, and maintain the balance between stability and mobility (19). Hence, bone density and strength are often cited as the primary indicators of normal bone formation. BMD actually reflects the amount of minerals (mainly calcium and phosphorus) in bone tissue, whose measurement usually relies on dual-energy X-ray absorptiometry (DEXA) (20). Low BMD correlates significantly with the increased risk of osteoporosis; thus, maintaining proper bone density is key to preventing osteoporosis and fractures. Bone strength, in contrast, is not only dependent on BMD but is also associated with the structure and quality of the skeleton, as monitored by more multifaceted parameters such as bone volume fraction (BV/TV), trabecular number (Tb. N), connectivity density (Conn. D), and trabecular thickness (Tb. Th) (21). However, the criteria for bone metabolism are more detailed, which include substances such as osteocalcin (OC; promotes bone production) (22), C-terminal telopeptides of type I collagen (CTX; suggests the activity of bone resorption), type I pre-collagen carboxy-terminal pre-peptide (P1CP; monitors osteogenesis levels), type I pre-collagen amino-terminal pre-peptide (P1NP; reflects the amount of collagen in the bone), 1,25-dihydroxyvitamin D3 [1,25-(OH)_2_-D3; promotes the transportation of calcium to bone], parathyroid hormone (PTH; lowers bone mass and raises blood calcium), alkaline phosphatase (ALP; typically 50–130 U/L for women and 45–125 U/L for men), calcium (standardized value: 2.25–2.75 mmol/L), and phosphorus (standardized value: 0.97–1.61 mmol/L) (23). In addition, the genetic, hormonal, and nutritional levels of individuals should not be overlooked in the evaluation of bone quality and quantity (24).

Abnormalities in bone mass directly contribute to osteoporosis, whose occurrence is mainly attributed to insufficient bone mass accumulation in early life and accelerated bone loss in later life. Early bone mass accumulation refers to bone mineralization that occurs during the younger years of individuals (primarily childhood and adolescence), which determines the overall health of the skeleton (25). Osteoporosis involves a gradual loss of minerals (especially calcium) with age (26). Altogether, the accumulation and loss of the bone are involved in establishing skeletal foundation, maintaining bone microstructural stability, and improving fracture resistance of the skeleton, thus inhibiting osteoporosis, particularly in the hip and spine. Optimizing peak bone mass (PBM) may be a proven solution (27), which denotes the total amount of bone tissue that an individual reaches during the most mature period of body growth (usually at 20–30 years old). The level of PBM and the cumulative rate of bone loss due to menopause and aging determine the possibility of developing osteoporosis. It has been reported that a one standard deviation increase in PBM lowers lifetime fracture risk by 50% (28). Lifestyle modifications centered on nutritional intake (calcium and vitamin D) and moderate exercise are known to be effective in enhancing PBM, thereby preventing osteoporosis and ameliorating prognosis (29). In addition, the first-line medications for patients with osteoporosis consist of bisphosphonates (BPs), selective estrogen receptor modulators, parathyroid hormone analogs, and therapeutic doses of calcium and vitamin D, which improve bone quality by inhibiting bone resorption, promoting bone formation, and supplementing bone nutrition (30). BPs, in particular, are vital in inhibiting osteoclast activity and decelerating bone loss by specifically binding to skeletal hydroxyphosphatidylcholine, thereby lowering blood calcium concentrations and reducing the incidence of osteoporosis (31). In addition to vitamin D3, which promotes the absorption of calcium and phosphorus in the intestine, its active metabolite, osteotriol, can also directly participate in bone mineral metabolism without hepatic or renal hydroxylation (32). However, even with the support of evidence-based medicine and extensive applications in a clinical setting, first-line medications for osteoporosis still fall short in terms of patient adherence, gaps in drug efficacy compared to trial outcomes, side effects, drug selection, and personalized treatment (33).

As early as 1980, patients who received total parenteral nutrition for more than 3 months were reported to suffer from orthopedic diseases such as ostealgia and osteochondrosis. With the empirical evidence of intestinal factors affecting osteoporosis, the mechanisms of how the intestinal intrinsic structure, gut microbiota, and microbial metabolites interact with bone health have been increasingly elucidated. As mentioned above, osteoporosis shows significant individual variation due to internal/external influences such as genetics, hormones, age, and diet, and the gut acts as a natural dynamic lumen exposed to the external environment, forming the basis for the study of the gut–bone correlation (34). The intestinal lamina propria is composed of the mucosa, submucosa, muscularis propria, and plasma, among which the mucosa is located in the innermost part of the intestinal wall and consists of the epithelium, lamina propria, and mucosal muscularis propria (35). The intestinal epithelium is arranged in a single layer of columnar cells embedded with tight junction proteins [Claudin, Occludin, and Zonula occludens (ZO)] (36), while immunocytes, such as lymphocytes, plasma cells, and macrophages, are present in the lamina propria and participate in intestinal immune defense (37). Between the intestinal lumen and feces is the mucus layer, where most of the gut microbes and their derived metabolites reside. The intestinal microbiome consists mainly of microorganisms such as bacteria, fungi, and viruses, numbering up to 100 trillion, with more than 1,000 species, which maintain an organized equilibrium in an uninterrupted exchange of material and energy with the organism (38). A large number of bacterial metabolites are produced in the process, including short-chain fatty acids (SCFAs; such as acetic and propionic acids), secondary bile acids (SBAs), neurotransmitters [such as gamma-aminobutyric acid (GABA), dopamine (DA), serotonin (5-HT), histamine (Hi), and glutamic acid (Glu)], and other substances such as indoles, branched-chain amino acids (BCAAs), and vitamins (39). In terms of gut–bone synergy, the mechanical barrier formed by the triad of intestinal mucosal epithelial cells, intercellular tight junctions, and bacterial membranes prevents pathogen invasion and toxin infiltration (40). Various derived metabolites such as SCFAs, SBAs, 5-HT, Hi, and vitamins are more often indicated as signaling molecules and substrates of metabolic reactions, which affect the distal bone tissues through pathway conduction and cascade, and participate in the formation, metabolism, and maintenance of the skeleton (41).

**Figure 1:**
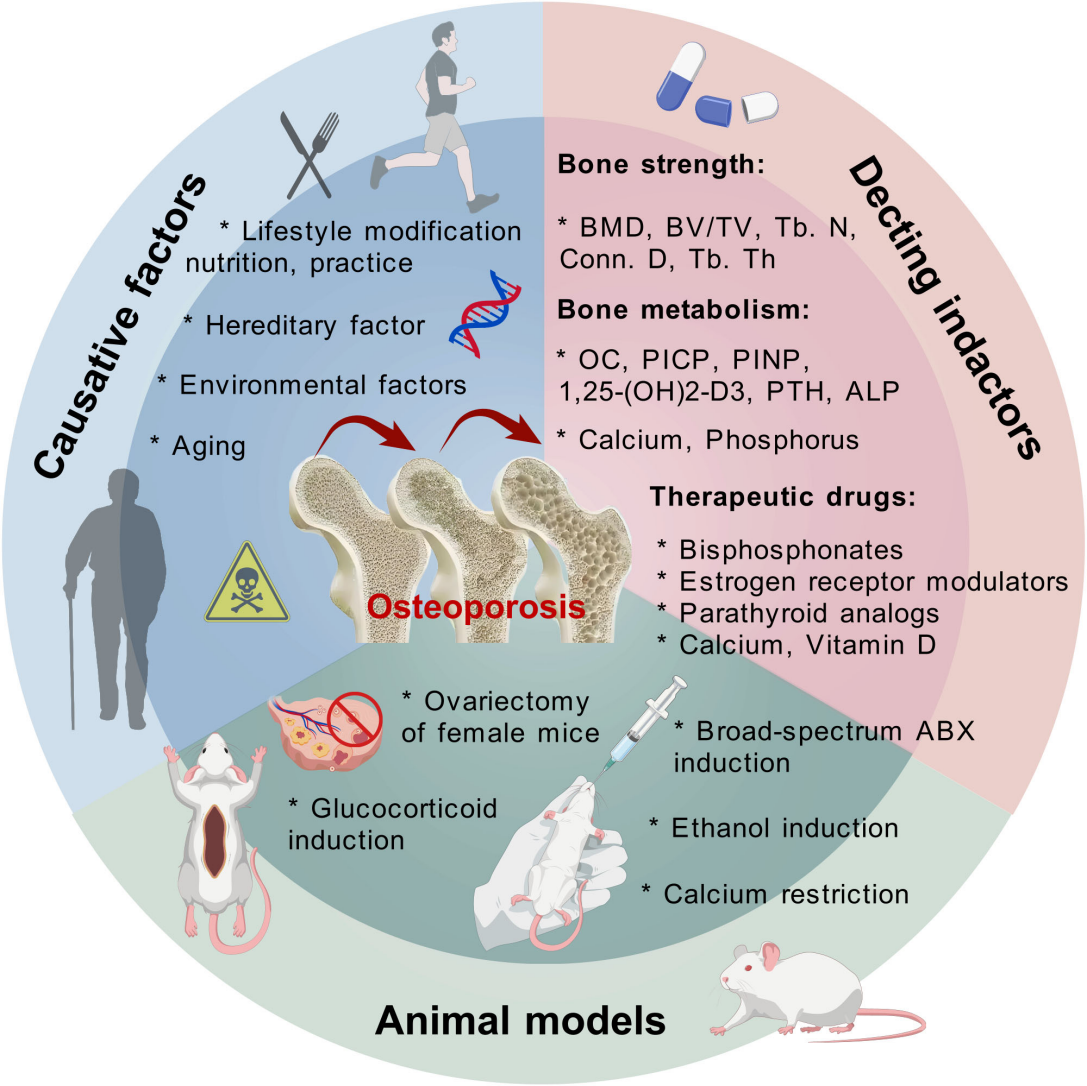
The causative factors, detection indicators, therapeutic drugs, and construction of experimental animal models of osteoporosis.

Hence, the concept of the gut–bone axis emerged. Current research on the role of the gut–bone axis in osteoporosis mainly targets the regulation of bone metabolism by intestinal factors, the pathogenesis and therapeutics of osteoporosis, and the interaction of the gut–bone axis with other systems. To thoroughly investigate the underlying associations, several types of animal models have been used. Among them, the PMOP mouse model simulated by ovariectomy is the most versatile (42), and exogenous glucocorticoids administered at high doses over a long term also induce similar osteoporotic manifestations in C57BL/6 female mice (43). Consistent with the etiology of osteoporosis, the effect of bone mass accumulation profile on skeletal health in early adulthood has also been replicated in experimental animals. Xi X et al. (44) employed 7-week-old male Wistar rats of normal growth, while Yuan Y et al. (45) constructed an early bone mass deficiency model using calcium-restricted Sprague–Dawley (SD) rats. Interestingly, bone loss was observed in 12-week-old male BALB/c mice after broad-spectrum antibiotics (ABX) treatment for 2 weeks (46), and an alcoholic osteoporosis model induced by ethanol has also been established to counteract the skeletal problems in chronic drinkers (47). In clinical practice, randomized controlled trials (RCTs) were widely conducted to evaluate the efficacy of fecal microbiota transplantation (FMT), probiotics, prebiotics, and other interventions that target the gut–bone axis for osteoporosis, providing more feasibility for the integrated management of osteoporosis through the analysis of intestinal microbial data, BMD, and bone metabolic markers (48). Regarding diagnostic tools, high-throughput sequencing, predominantly 16S rRNA amplicon sequencing (49), and the integration of multi-omics technologies (including genomics, transcriptomics, proteomics, and metabolomics) (50) have enabled a more comprehensive advancement in exploring the mechanisms by which the gut microbiota and related molecules exert their anti-osteoporosis effects (**Figure 1**).

## 3 Holistic account of the mechanism of gut–bone axis and molecules involved

### 3.1 Direct effect of gut factors on bone health

#### 3.1.1 Microbe-derived components in bone homeostasis

Intestinal factors act on bone either directly or through immune and endocrine transitions, with the direct role of SCFAs in influencing osteoclasts and osteoblasts being prominent. As the principal products of dietary fiber fermented by intestinal microbiota, SCFAs such as acetic, propionic, and butyric acids can provide energy to enterocytes and promote intestinal barrier integrity (51). In molecular signaling, SCFAs activate the members of the G protein-coupled receptor (GPR) family, such as GPR41, GPR43, and GPR109A, which in turn regulate the key pathways of bone metabolism, Wnt/β-catenin, and the receptor activator of nuclear factor-κB ligand (RANKL)/RANK/osteoprotegerin (OPG), for instance (52). When Wnt signaling is activated, β-catenin accumulates in the cytoplasm and enters the nucleus, where it binds to T-cell factor (TCF)/lymphoid enhancer factor (LEF) transcription factors and promotes the expression of osteogenesis-related genes, thereby increasing bone formation (53). For the RANKL/RANK/OPG pathway, RANKL/RANK binding promotes the differentiation and activity of osteoclasts, while OPG acts as a decoy receptor for RANKL to inhibit the above process (54). RANK also serves as an activator of the nuclear factor κB (NF-κB) receptor, which is likewise involved in osteoclast differentiation (55). Additionally, SCFAs mediate bone mineralization and bone formation through the runt-related transcription factor (RUNX) signaling pathway, a key factor regulating the differentiation of osteoblast marker genes, which induces the directional differentiation of bone marrow stromal cells to bone and cartilage precursor cells (56).

In addition to SCFAs, how other microbial-derived molecules directly affect bone health cannot be neglected either. The gut microbiota metabolizes tryptophan (Trp) to two types of products, indoles and quinolinic acid (QUIN) (57). As an endogenous ligand for the intestinal aryl hydrocarbon receptor (AhR; a transcription factor), indole-acrylic acid (IA), indole-3-propionic acid (IPA), and indole-acetic acid (IAA) can achieve the specific activation of AhR (58). AhR signaling not only directly inhibits the differentiation of osteoclasts induced by RANKL but also inhibits the NF-κB pathway in osteoblasts and promotes the expression of genes related to osteogenesis, such as Runx2 and Osterix (59). QUIN has been reported to mediate mitochondrial oxidative stress through the activation of the *N*-methyl-d-aspartate receptor (NMDAR) (60), yet its studies on bone maintenance remain lacking. Regarding the intrinsic structure of intestinal bacteria, lipopolysaccharide (LPS) is a major component within the cell wall of gram-negative bacteria (61). When intestinal microecology is disrupted, LPS enters blood circulation, activates the Toll-like receptor 4 (TLR4) signaling, and upregulates the expression of the NF-κB pathway, which in turn promotes the differentiation and activity of osteoclasts, leading to severe bone loss and osteoporosis (62).

#### 3.1.2 Intestinal permeability in maintaining bone health

The intestinal mucosa acts as a selective filter, controlling material passage, mainly co-regulated by tight junction proteins and the mucus layer (63). Abnormal intestinal permeability leads to the endocytosis of bacterial endotoxins, pathogens, and undigested particles known as leaky gut (64), especially the LPS-TLR4/NF-κB pathway, which develops a direct stimulus to osteoclasts (65). A clinical study enrolling 150 IBD patients also provided empirical evidence for the significant correlation between leaky gut and decreased BMD (66). It is worth stating that the gut microbiota and their derived metabolites are critical in maintaining normal intestinal permeability. Beneficial bacteria such as *Lactobacillus* and *Bifidobacterium* secrete antimicrobial peptides (AMPs) to inhibit the intestinal colonization of pathogenic bacteria and maintain intestinal barrier integrity (67). SCFAs inhibit histone deacetylase (HDAC) and activate the adenosine 5′-monophosphate-activated protein kinase (AMPK) pathway, which in turn promotes the expression of ZO-1 and Occludin (68). Chen C et al. (69) also observed that supplementation of Trp metabolites such as IAA and IPA repaired the intestinal barrier integrity and bone loss in PMOP mice in an AhR-dependent manner, which involved the crosstalk between AhR and Wnt/β-catenin signaling cascade (70). The above elaborations support the crucial role of intestinal permeability in the gut microbiota–bone axis.

### 3.2 Gut–immune–bone axis: indirect effects through immunization

#### 3.2.1 Participation of diverse immunocytes in osteoporosis

As a chronic disease, the mechanisms of osteoporosis cannot be separated from immunity and inflammation; hence, the activation of immunocyte clusters and inflammatory cytokines that are highly correlated with bone health was explored. The first are Treg and Th17 cells, as subpopulations of lymphocytes with opposite functions, whose balance is essential for inhibiting osteoclast activity and maintaining bone homeostasis (71). Th17 releases interleukin-17 (IL-17) and stimulates osteoblasts and mesenchymal stem cells (MSCs) to express RANKL, thereby accelerating the differentiation of osteoclasts (72). In contrast, IL-10 and transforming growth factor-β (TGF-β) secreted by Treg display exactly the opposite effect. It is worth emphasizing that TGF-β, as a regulatory cytokine, participates in the activation of the bone morphogenetic protein (BMP)/Smad signaling pathway while mediating inflammation, which in turn regulates the differentiation and proliferation of osteoblasts, hence facilitating bone formation and restoration (73). The internal antagonism of macrophages likewise contributes to skeletal immune homeostasis. M1 macrophages secrete pro-inflammatory factors such as tumor necrosis factor-α (TNF-α) and IL-1β to inhibit osteoblast proliferation and promote osteoclast differentiation, while anti-inflammatory M2 macrophages are beneficial to bone restoration (74). Moreover, certain activated B lymphocytes have also been reported to communicate with osteoblasts and osteoclasts *via* molecules including RANK, IL-7, and anti-OPG antibodies (75).

#### 3.2.2 How gut factors regulate bone health *via* immune system

Except for the direct targeting of osteocytes as described above, intestinal factors also influence the distal bone tissue through the immune and inflammatory pathways. Going back to SCFAs, butyrate, propionate, and acetate were observed to possibly induce the expression of forkhead box protein 3 (Foxp3) to trigger the development of Treg while inhibiting CD4^+^ T cells (76). Trp metabolism in the intestine, as well as exogenous IAA and IPA, enhances M2 macrophages and bolsters the massive diffusion of IL-10 from the intestinal lamina propria to the bone marrow, thereby significantly promoting osteogenesis (70). While anchoring osteoclasts, LPS also activates the TLR4 signaling on the surface of macrophages, prompting the secretion and aggregation of cytokines such as TNF-α and IL-6 (77). The generation of anti-OPG antibodies in B lymphocytes was also dependent on leaky gut-induced entry of enterogenic antigens into the circulation, which in turn interferes with bone homeostasis (75).

### 3.3 Gut–endocrine–bone axis: intervention of hormones and other endocrine substances

#### 3.3.1 Endocrine hormones related to both the gut and the bone

The endocrine pathway is another indirect form of the gut–bone axis, in which the involvement of different types of hormones ranks as the most important aspect. As an endocrine cell distributed at the intestinal epithelium, enterochromaffin cells are capable of delivering 5-HT, also known as serotonin, which exerts a delicate and complex physiological effect on bone remodeling (78). As an important neuroendocrine mediator, 5-HT routinely travels through the bloodstream to the skeletal tissues, where it binds to appropriate receptors and regulates bone formation and resorption (79). However, high levels of 5-HT for a long period may lead to elevated osteoclast activity and bone resorption, which in turn result in osteoporosis. Other metabolic hormones also interact extensively with the gut microbiota and are involved in bone microecology. For example, intestinal β-glucuronidase-yielding bacteria such as Clostridia and *Bacteroides* catabolize the metabolized estrogen into a deconjugated active form, reabsorbing it through the enterohepatic circulation (80). The serum estrogen then binds to estrogen receptor (ER) α/ERβ in bone tissue, inhibiting the differentiation of osteoclasts through the RANKL/OPG signaling axis (81). PTH promotes bone formation by driving the differentiation of osteoblasts and coordinating the recruitment of osteoblast progenitor cells (82). However, when intestinal barrier damage leads to decreased calcium absorption, the insufficient concentration of blood calcium directly stimulates the parathyroid glands to secrete PTH, liberating calcium at the expense of bone resorption (83). Calcitonin secretion is similarly regulated by intestinal barrier function, which inhibits osteoblasts and promotes osteogenesis (84). After the intestinal epithelium absorbs amino acids such as leucine, mechanistic target of rapamycin complex 1 (mTORC1) signaling is activated to initiate the synthesis of insulin-like growth factor-1 (IGF-1) in hepatocytes (85). IGF-1 not only promotes cartilage formation and midshaft bone growth but also affects the progression of osteoblasts and osteoclasts (86). Interestingly, LPS leakage also exhibits endocrine disruption, causing abnormal cortisol release and accelerated osteoblast apoptosis by affecting the hypothalamic–pituitary–adrenal (HPA) axis (87).

It is well known that the biogenesis of bone is highly dynamic, with the process of bone resorption showing evident circadian rhythmicity (88). Previously, rhythmic signals sent from the center were believed to influence skeletal growth, metabolism, and homeostasis, whose disruption is significantly associated with decreased BMD (89). Based on the equally high sensitivity of intestinal physiology to circadian rhythm disruption, coupled with the influence of intestinal factors on bone remodeling, Ko FC et al. (90) identified colon epithelial cell-specific Bmal1, a clock gene that regulates the intestinal circadian rhythms and whose disruption was shown to result in the absence of bone trabeculae and cortex in male mice. Furthermore, skeletal circadian rhythms have been observed to be similarly regulated by a variety of gut hormones, including glucagon-like peptide-1 (GLP-1), glucagon-like peptide-2 (GLP-2), glucose-dependent insulinotropic polypeptide (GIP), and polypeptide YY (PYY) (91). In particular, GLP-1 and PYY, which are produced by enteroendocrine L cells, not only regulate the circadian rhythms of bone resorption and formation but also influence processes such as insulin secretion, appetite, and energy metabolism, which indirectly contribute to bone health.

Melatonin (MLT), a crucial endogenous hormone whose synthesis takes Trp as raw material, was also observed to influence bone health while linking with intestinal microecology. Through shared precursors, metabolic interactions, and functional collaborations, MLT and intestinal Trp metabolites constitute a classic host–microbial co-metabolic network (92), which has also been reported to have certain anti-osteoporosis effects. Chen Y et al. (93) observed that disturbed intestinal Trp metabolism due to osteoporosis exposure further triggered a decrease in gut microbiota-derived MLT, whereas the administration of MLT alleviated osteoporosis symptoms and reversed intestinal dysbiosis, like increasing the relative abundance of probiotics such as *Allobaculum* and *Parasutterella*. The acting spectrum of gut microbiota-derived MLT also involves the production of SCFAs and the downregulation of trimethylamine *N*-oxide (TMAO)-related metabolites, which precisely rehabilitates the disrupted intestinal microecology. Thus, microbial Trp metabolites are expected to be a feasible intervention site to inhibit osteoporosis progression through the gut–endocrine–bone axis. As a substance competing with intestinal Trp metabolism for precursors, MLT also participates in the dynamic modulation of M1/M2-type macrophage balance (94), which in turn reduces serum pro-inflammatory cytokine levels and restores intestinal barrier function.

#### 3.3.2 Vitamins absorbed through the intestine that act on bone

**Figure 2:**
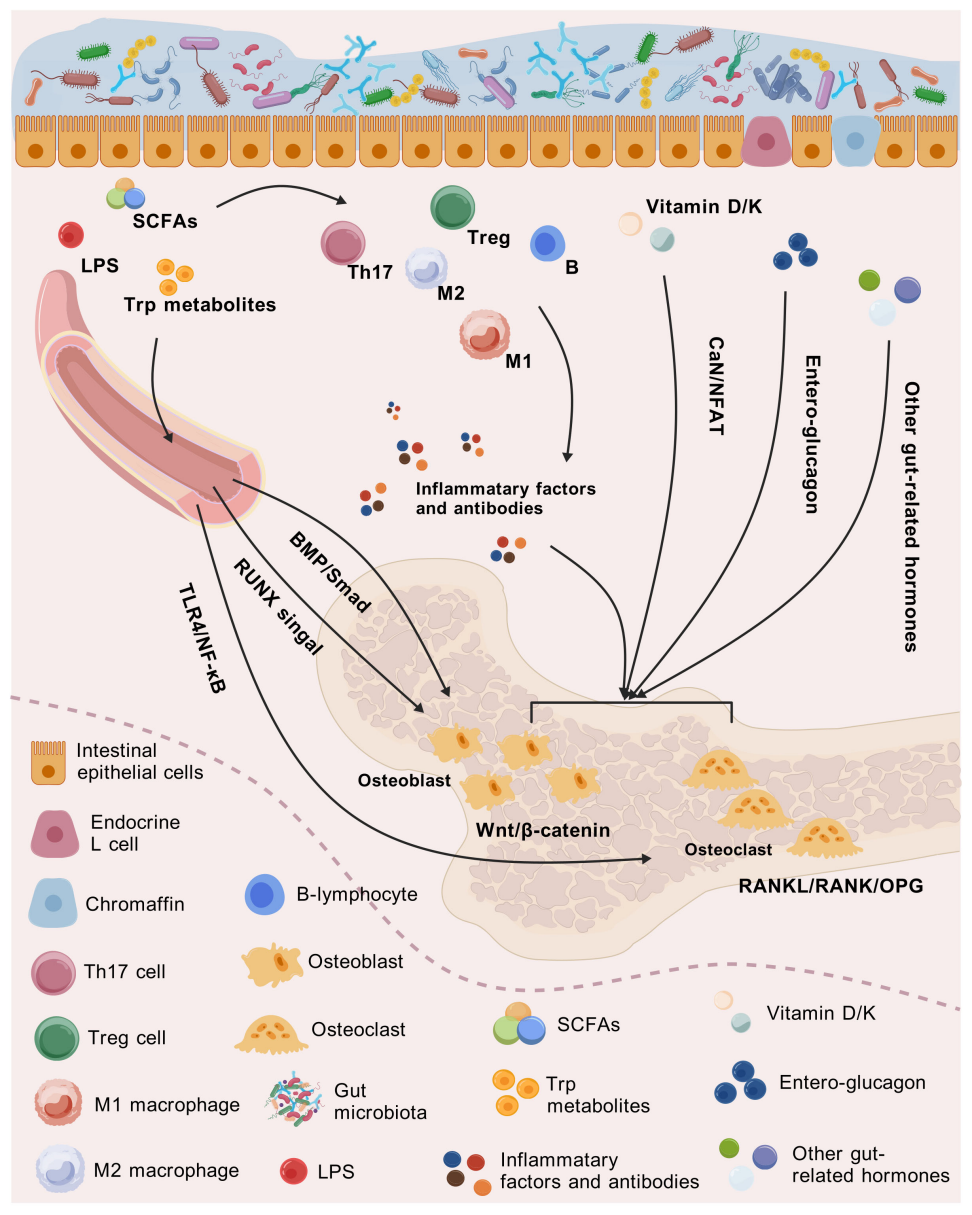
Mechanisms of how intestinal-related factors influence bone health through the gut–immune/endocrine–bone axis.

While vitamin D at therapeutic doses was mentioned earlier as an effective nutritional supplement for the bone, dietary intake of vitamin D also plays an essential role in maintaining bone homeostasis. The small intestine absorbs the liposoluble vitamin D, which is then activated to 1,25-(OH)_2_-D by the liver and kidneys, thereby stimulating calbindin in the intestinal epithelial cells to maintain adequate blood calcium concentration (95). Based on the calcium dependence of calcineurin (CaN), calcium further serves as a second messenger to drive the CaN/nuclear factor of activated T cells (NFAT) pathway, participating in osteoblast proliferation, differentiation, and apoptosis (96). In addition to absorbing dietary vitamin K, intestinal bacteria harboring the *Men* gene cluster can directly synthesize vitamin K2 *via* the menaquinone pathway. The obtained vitamin K operates as a coenzyme of γ-glutamyl carboxylase and engages in the carboxylation of bone matrix proteins such as osteocalcin, which is critical for maintaining bone strength and preventing osteoporosis (97) (**Figure 2**).

## 4 Strengthened concept of gut–bone axis: an exploration of gut–bone axis plus X

### 4.1 Extension on the boundary of gut–bone axis

Since the gut and bone are physically distant but closely interconnected, certain upstream, midstream, and downstream mediators, as well as other targets, are bound to exist to guarantee the integral harmonization of the organism’s activities, theoretically. Here, we propose a reinforced mode of the gut–bone axis plus X that the gut–bone axis synergizes with other tissues or organs in the form of additive effects on clinical manifestations and mechanisms of action that are in tandem, constructing a broader biological network at the core of the gut–bone axis while spreading to other systems of the organism. Regarding the form of characterization, it can be interpreted that the effect chains of the entero-skeletal axis may influence the genesis and progression of certain systemic pathologies, or the presence of complications in specific diseases may be significantly linked to the gut–bone axis.

#### 4.1.1 Gut–bone axis and the hematopoietic system

Bone tissues, except for the skeleton, as the most vital sustaining structure of the human body, the red bone marrow in long bones, and the cancellous stroma in flattened bones, also possess a lifelong hematopoietic function (98). In the bone marrow, the biogenesis of hematopoietic stem cells to hemocytes, leukocytes, and platelets maintains the homeostasis of the blood components to a large extent (99). Thus, the interaction between the gut–bone axis and the hematopoietic system is the chief problem. One study reviewed the impact of the gut–bone axis in the context of aging on phenomena constituting osteoporosis and hematopoietic hypoplasia (100). It noted that the key point would be the communication between gut-related agents [SCFAs, lactate, tryptophan metabolites, iron potency, bacterial extracellular vesicles, TLR signaling, and microbe-associated molecular patterns (MAMPs)] and hematopoietic-related immune cells, to which these kinds of aging-related functional degeneration should be attributed.

#### 4.1.2 Gut–bone–tumor axis

Interestingly, there was a study reporting the involvement of the gut–bone axis in breast cancer (BC) and proposing the concept of the “gut–bone-tumor axis” innovatively. Mechanistically, BC is a kind of tumor that is greatly affected by endocrine disorders. The levels of hormones such as estradiol and progesterone are highly correlated with the incidence of BC, while obesity resulting from improper diet is also one of the pathogenic factors that cannot be ignored (101), which corresponds to the gut–bone axis. Therefore, it is of clinical significance to describe the gut–bone axis in BC. Chen J et al. (101) demonstrated that intestinal dysbiosis induced by high-fat diet promotes the release of leucine into the peripheral bloodstream, which further activates the rapamycin complex 1 (mTORC1) signaling in medullary progenitor cells, triggers the differentiation of polymorphonuclear myeloid-derived suppressor cells (PMN-MDSCs) (102), and ultimately results in the adverse clinical outcomes of BC. Concentrating on the “gut–bone–tumor axis”, that study provides unique insights for BC management, on the one hand, and paves novel avenues for anticancer therapies targeting the intestinal dysbiosis, on the other hand. Nevertheless, it is worth stating that the exploration of more tumor types and more comprehensive mechanisms that correlate with the gut–bone axis is absolutely necessary; otherwise, the “gut–bone–tumor axis” may remain in the awkward conceptual stage.

#### 4.1.3 Gut–bone axis and diabetes

According to the gut–endocrine–bone axis described above, GIP and GLP-2 are involved in the maintenance of bone homeostasis as regulators of bone conversion. As key enteroglucagons, GIP and GLP-2 themselves exhibit excellent hypoglycemic properties (103), which may be the rationale for the link between the gut–bone axis and diabetes. Several studies on type 1 diabetes mellitus (T1DM) and type 2 diabetes mellitus (T2DM) coincide with our conjecture. On the premise that T2DM patients with normal BMD still present an increased risk of fracture, Skov-Jeppesen K et al. (104) injected exogenous GIP and GLP-2 subcutaneously into 10 male T2DM patients. At the endpoint of the trial, significant reductions in several indices related to bone conversion, such as CTX, P1NP, osteosclerotic proteins, and parathyroid hormones, were observed compared with the placebo and baseline data. The above results organically combine the gut hormones and bone conversion together to achieve effective control of the elevated fracture risk in diabetes. In addition to T2DM, Hartmann B et al. (105) obtained consistent conclusions in T1DM. This observational case–control study targeting T1DM verified that patients with T1DM showed significantly impaired inhibition of bone resorption (as assessed by CTX) and bone formation (as assessed by P1NP) in oral glucose tolerance test (OGTT), in comparison to the iso-glycemic intravenous glucose infusion (IIGI) and healthy controls. Interestingly, this phenomenon was found to be independent of insulin secretion and glucose fluctuations, which further reflects the influence of the gut–bone axis and enteroglucagons on reduced BMD and increased fracture risk in diabetic patients.

#### 4.1.4 Gut–bone axis and periodontitis

Periodontitis is a chronic infectious disease caused by periodontal pathogenic bacteria, which stimulates the differentiation of osteoclasts while inducing periodontal inflammation, resulting in alveolar bone resorption (106). In addition to the oral microbiota disturbance, intestinal microecology contributes as well. Studies have reported that PMOP plays a promoting role in the genesis and progression of periodontitis, which is legitimately associated with systemic bone destruction accompanied by PMOP, including the alveolar bone. To figure out whether the process of PMOP leading to periodontitis explicitly involves the presence of the gut microbiota or the gut–bone axis, Han N and Jia X et al. (107, 108) developed a multifaceted elaboration in the aspects of circulatory metabolism and immune mediation that related to the gut–alveolar bone axis. On the one hand, intestinal bacterial compositions such as LPS and SCFAs may distally transfer to the alveolar bone to regulate bone homeostasis. On the other hand, intestinal pathogenic bacteria induce the homing of immunocytes such as Th1, Th17, and natural killer (NK) cells to distant organs, leading to pathological alveolar bone loss. In addition, gut dysbiosis enhances systemic inflammation and subsequently releases cytokines such as IL-6 and TNF-α, which in turn exacerbate periodontitis. Hence, it can be implied that anti-osteoporosis strategies targeting the gut–bone axis may possess an adjunctive role in the prevention of periodontitis complicated by PMOP.

#### 4.1.5 Brain–gut–bone axis

The human body is functionally interconnected as a whole, and thus, synergies between the gut–bone axis and other organs in terms of mechanisms are also predictable. In the gut plus X regime, the brain–gut axis is a well-deserved hotspot. This bidirectional signaling between the brain and the intestine is essentially in tandem in the neural, endocrine, and immune pathways (109). In particular, neuroendocrine conduction, with neurotransmitters such as acetylcholine (ACh) and GABA directing the connection between the brain and intestine *via* the vagal and spinal nerves, plays a role in regulating the sensory, motor, and secretory functions of the digestive system (110). The gut–brain axis is also inseparable from the HPA axis, a crucial signaling cascade system. Corticotropin-releasing hormone (CRH) and arginine vasopressin (AVP) secreted by the hypothalamus act on the anterior pituitary and accelerate the release of adrenocorticotropic hormone (ACTH) and its stimulation of the adrenal cortex, further triggering the secretion of cortisol and other hormones and ultimately reaching the target site through blood circulation, affecting the functional and immune states of the intestinal tract (111). The reported presence of hypothalamic neuroendocrine alterations in PMOP patients laid a factual foundation for the research of the brain–gut–bone axis. Furthermore, Chen Z et al. (112) verified the negative regulation of neuropeptide Y (NPY), a potential neuroendocrine signal, on the brain–gut–bone axis in ovariectomy (OVX) rats. Under the condition of exogenous NPY overexpression, bone formation and bone microarchitecture were disrupted, pyroptosis-related molecules such as NOD-like receptor protein 3 (NLRP3) and Caspase-1 in subchondral cancellous bone were upregulated, and the levels of colonic inflammatory indicators like IL-1β, IL-18, and serum LPS were elevated as well. Correspondingly, the altered diversity and composition of the gut microbiota were observed, including α-diversity represented by the Shannon, Simpson, and Chao1 indices; β-diversity assessed by principal coordinate analysis (PCoA); the ratio of Firmicutes to Bacteroidetes (F/B); and the relative abundance of Clostridia, Bacteroidia, and Lachnospirales. Y1R antagonist (Y1Ra; blocking NPY receptor) and FMT from healthy samples alleviated or even reversed the above alterations to some extent, suggesting the possibility of targeting the NPY-mediated brain–gut–bone axis for PMOP intervention. In addition, Zhang YW et al. (17) also reviewed the regulatory effects of probiotics on bone metabolism through the brain–gut–bone axis, stating the underlying mechanisms by which physicochemical factors, such as the intestinal epithelial barrier and derived metabolites, influence osteoporosis through neurological, immunological, and endocrine factors.

#### 4.1.6 Gut–liver–bone axis

As emphasized above, the activation of vitamin D from intestinal sources depends on the support of the liver. Analogously, studies on the gut–liver–bone axis have elaborated on the adjunctive role of the liver in the gut–bone axis, with BA metabolism as an indispensable intermediary. Carson MD et al. (113) simulated the bone formation during puberty on specific pathogen-free (SPF) and germ-free (GF) mice at 6–12 weeks by administering a therapeutic dose of minocycline (a systemic ABX indicated for juvenile acne) and explored the involvement of the gut–liver–bone axis in osteoporosis pathogenesis. As described, ABX-induced intestinal dysbiosis results in the altered microbial BA metabolism, which destroys the homeostasis between BA synthesis in the liver and BA metabolism in the intestine, restricts the farnesoid X receptor (FXR)/fibroblast growth factor 15 (FGF15) signaling axis in enterocytes with the conjugated BAs being an effective inhibitor of FXR, and further undermines the interplay of FGF15 with fibroblast growth factor receptor 4 (FGFR4) in the hepatocytes (114). The mutual outcome of these two effects is the upregulation of hepatic BA formation and emission of conjugated BAs into the blood circulation, which distally anchors the FXR in the bone marrow, resulting in the loss of osteoblast function, negatively impacting BMD, bone volume fraction, and fracture resistance (115).

### 4.2 Refinement on the connotation of gut–bone axis

**Figure 3:**
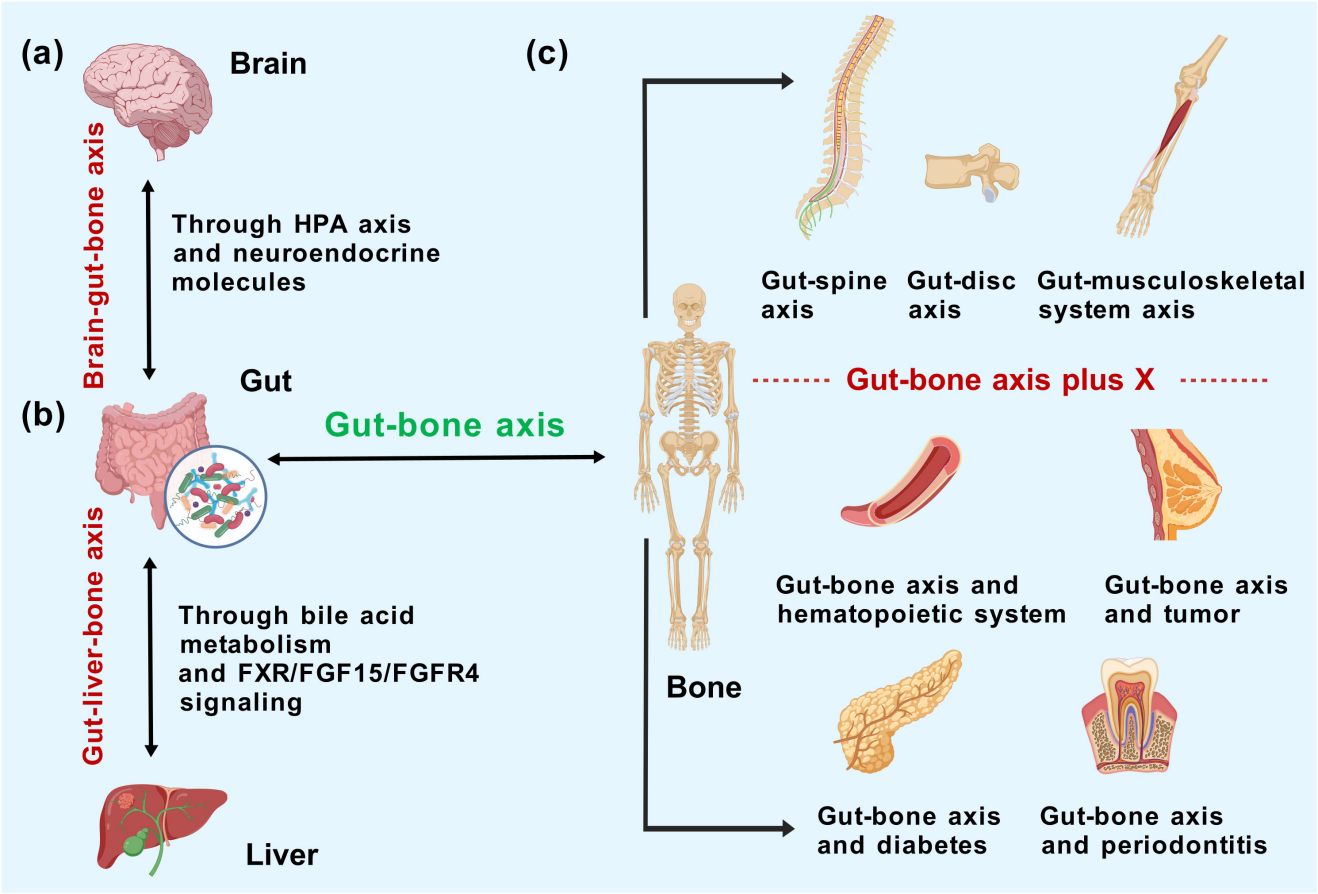
Mechanistic correlation of the gut–bone axis with **(a)** brain and **(b)** liver and **(c)** the concept extrapolation/shrinkage of gut–bone axis according to representational relevance.

In the downscaled concept of the gut–bone axis, the influence of gut microbes on the entire skeletal system is de-emphasized; here, we mainly summarize the role of the gut microbiota in specific parts of the skeleton (such as the femur and vertebrae). A Mendelian randomization conducted by Chen S et al. (116) to clarify the causality between the gut microbiota and musculoskeletal disorders restricted the scope of orthopedic pathologies to six musculoskeletal diseases that may be highly associated with the gut–bone axis: osteoporosis, fracture, myasthenia gravis, low back pain (LBP), rheumatoid arthritis (RA), and ankylosing spondylitis. The inverse variance weighting (IVW) method in combination with Bonferroni correction ultimately yielded the causal relationships between genus *Bifidobacterium* and elevated left handgrip strength, genus *Oxalobacter* and the high risk of LBP, and family Oxalobacteraceae and the decreased risk of RA. The above results were obtained from the genetic level, which laid a foundation for further basic experiments and clinical validation. The concept of the gut–spine axis and gut–disc axis is even narrower. Morimoto T et al. (117) proposed that the degeneration of spinal components is associated with gut microbiota anomalies and separately categorized osteoporosis, synovial osteoarthritis, disc degeneration, spinal sarcopenia, and lumbar stenosis under spinal degenerative diseases (SDD), introducing the concept of the gut–spine axis for the first time. Based on the primary etiology and location of LBP, Li W et al. (118) initially constructed the gut–disc axis to summarize the overall impact of gut microbiome on intervertebral disc degeneration (IDD). The results suggested that strategies targeting the gut microbiota and interrupting the gut–disc axis cascade can inhibit the inflammation in IDD and thus alleviate LBP. Indeed, investigating a relatively narrow but centralized concept can simplify the workflow and help draw parallels to guide the interventions for a collective group of orthopedic diseases. However, the desirability of the above axes depends, in principle, on the theoretical and practical implications of characterizing some different orthopedic diseases within a single context; otherwise, it would remain just a vague definition (**Figure 3**).

## 5 Strategies for osteoporosis management: gut–bone axis targeted

### 5.1 FMT, probiotics, and prebiotics

#### 5.1.1 FMT

As a novel technique of “organ transplantation”, FMT involves transferring fecal microbiota from healthy donors to recipients to modify disturbed intestinal ecology, whose efficacy in treating digestive diseases such as *Clostridioides difficile* infection (CDI) (119), ulcerative colitis (120), and IBS (121) has been repeatedly verified. The effect spectrum of FMT has also taken a qualitative leap in recent years, with the paving of networks such as the gut–brain axis, gut–liver axis, and the intestinal–vaginal bi-luminal tract. Hence, diseases like Parkinson’s disease, alcoholic fatty liver, and epithelial ovarian cancer, which affect multiple systems throughout the body, can be partially or substantially intervened by FMT. Similarly, both basic and clinical studies on FMT for bone health are underway, fueled by increasing empirical evidence of the gut–bone axis. Wang et al. (122) transferred feces from osteoporotic rats to young rats and observed the onset of osteoporosis in the recipients, which was primarily ascribed to gut microbiota dysbiosis and intestinal mucosal barrier damage. Atarashi et al. (123) also found that *Clostridium* IV and XIVa isolated from healthy mice increased the number of systemic and lamina propria Treg cells when transplanted into germ-free mice, which in turn affected osteoclast genesis through the secretion of IL-4, IL-10, and TGF-β. Moreover, a study (43) has reported bone loss in antibiotic-pretreated mice 1 week after receiving fecal bacteria from mice with glucocorticoid-induced osteoporosis, somewhat corroborating the endocrine involvement of FMT in influencing bone health. To summarize, FMT first exerts *in situ* effects on intestinal metabolites and the intestinal mucosal barrier, consequently working through the immune and endocrine pathways to regulate bone mass. Nowadays, the application of FMT in anti-osteoporosis treatment remains in the research stage and has not yet been applied in a clinical setting. Due to the lack of standardized treatment protocols, transplantation routes, donor selection, microbiota preparation, and storage methods may affect the ultimate results (124). Meanwhile, given the individual differences in gut microbiota composition and metabolic characteristics of osteoporosis patients, the risk of immune rejection and infection associated with long-term transplantation, and the ethical issues and informed consent related to the protection of donor–recipient privacy (125), there may still be a long way to go before the use of FMT in the management of osteoporosis.

#### 5.1.2 Probiotics

Probiotics are live microorganisms that benefit human health when consumed in adequate amounts (126). Somewhat unlike FMT, the administration of combined or single probiotics has been more recognized as alleviating digestive diseases and lesions distant from the intestinal tract, as well as in orthopedic diseases. Considering the altered gut microbiota in the context of bone loss as discussed above, the intake of probiotics from Firmicutes has become our major concern. Surprisingly, we observed the positive effect of a series of probiotics classified morphologically as bacilli on bone quality enhancement. The administration of *Bifidobacterium bifidum* FL228.1 and *Bifidobacterium animalis* subsp. *lactis* F1-7 (127) was reported to increase the relative abundance of beneficial bacteria such as *Lactobacillus* and *Colidextribacter* while inhibiting the colonization of pathogenic bacteria like *Desulfovibrio*. The remodeled microbiome composition further optimizes the integrity and permeability of the intestinal barrier *via* Occludin, ZO-1, Claudin-2, and Mucin 2 (MUC2), at the same time suppressing the activity of M1-type macrophages, thereby improving the clinical indices of bone health, including BMD, BV/TV, and Tb. N. Similar osteoporotic remission was also observed in OVX mice treated with quantitative gavage of *Rothia* for 8 weeks (128). In addition, *Lactobacillus rhamnosus* GG (129) strengthened bone microstructure and bone biomechanics by adjusting the ratio of Firmicutes/Bacteroidetes, maintaining Th17/Treg balance, repairing mucosal damage caused by estrogen deficiency, and upregulating the levels of GLP-2R, CTX, P1NP, and RANKL. A similar phenomenon was observed in heat-killed *Lacticaseibacillus paracasei* GMNL-653 (130), which was confirmed by *in vitro* and *in vivo* models and whole-genome sequencing. Herein, the mechanism of probiotics influencing bone health involves first exerting *in situ* effects on the gut microbiota and intestinal barrier, then affecting the interactive pathways of the gut–bone axis (including endocrine, inflammation, and immunity), and ultimately regulating bone homeostasis.

In the field of bacterio-pharmaceuticals, the preference for probiotics over FMT can be largely attributed to the fact that probiotics, as the most potent component of fecal microbiota, are better established in research, while the operation process is more streamlined and controllable. This norm seems to be equally applicable in the gut–bone axis in treating orthopedic diseases. While mono-bacterial supplementation facilitates the exploration of the mechanisms of the gut–bone axis and expands the insights into microbiome–host interactions, multi-bacterial combinations can be constantly optimized in terms of procedures and proportions to see if there are synergies or detractions, thus maximizing the efficacy of probiotics in the treatment of orthopedic disorders. Therefore, it is ideal to evaluate the efficacy of probiotics on orthopedic diseases under the premise of ethical approval.

#### 5.1.3 Prebiotics

In addition to FMT and probiotics, prebiotics have also been reported to treat orthopedic disorders through the gut–bone axis. Prebiotics can be defined as a non-digestible carbohydrate that selectively stimulates the activity of beneficial bacteria in the intestinal tract, thereby positively affecting host health (131). Certain strains in the gut microbiota ferment prebiotics and influence the health of the skeleton in a variety of ways, for instance, promoting the production of SCFAs, lowering luminal pH, improving the absorption of calcium and phosphorus, enhancing anti-inflammatory properties, and modulating intestinal Treg cells. The prospect of prebiotics such as oligofructose (FOS), galacto-oligosaccharide (GOS), inulin, and phenolic acids applied to support bone quality has been elucidated in several studies (29). Especially for FOS, a study specifically comparing FOS supplementation with blank control and tart cherry addition (TC; a natural active substance) verified in an osteoporosis model of C57BL/6 female mice that FOS significantly increased the content of SCFAs and the BMC, density, and BV/TV in vertebral bodies and the proximal tibia, which involved the presence of osteoblast genes (Wnt10b, Bmp2, Osterix, and Col1a1) and osteocyte genes (Phex, Dmp1, Mepe, Cnx43, and Sost). Interestingly, the above effects on osteoblasts and osteocytes were independent of Treg cells. Similarly, konjac oligosaccharides (KOSs) (71) were also observed to increase the number of bone trabeculae by 134.2% and bone bending stiffness by 103.1% in OVX rats, thereby significantly alleviating bone loss. This effect was achieved through the gut–bone axis (promoting the growth of *Bifidobacterium longum*, repairing intestinal mucosal barrier, inhibiting pro-inflammatory cytokines, and restoring Treg/Th17 balance in bone tissue), helping to inactivate the osteoclasts. Furthermore, researchers used resveratrol (132), a widely employed polyphenol with prebiotic properties that can be converted into a highly metabolically active molecule by the gut microbiota, thus serving as one kind of epigenetic modulator in the combined therapy and prevention of osteoporosis.

### 5.2 Dietary interventions

#### 5.2.1 Dietary supplements or nutrients

Parallel to prebiotics, some other dietary supplements or nutrients have also been shown to alleviate orthopedic diseases, whose function varies depending on the composition. Multiple different nutrients with bone health-promoting effects, such as dietary fiber (DF), vitamins, and ketones, were observed. The first is DF, a carbohydrate polymer derived from bacterial cell walls that cannot be hydrolyzed by enzymes in the small intestine, which exerts complementary or overlapping functions with prebiotics. Wu Y et al. (133) administered DF at different levels for 8 weeks in an SD rat model of osteoarthritis, and 16S rRNA sequencing showed that high DF intake significantly increased the abundance of Bacillota-dominant microbiota and attenuated the extent of osteoarthritic lesions. The latter was achieved mainly through the gut–bone axis by upregulating SESN2 expression in the knee joint, maintaining chondrocyte activity, and mitigating synovial inflammation. Another promising dietary supplement, menaquinone-7 (MK-7), presents a slightly different mode of effect (45). As the most bioavailable and stable congener of vitamin K2, one study evaluated the effects of exogenous intake of MK-7 combined with vitamin D on bone formation in SD rats growing under calcium limitation. The results showed that MK-7 supplementation improved several bone quality parameters, such as femoral cortical thickness, cortical bone area, and calcium content, on the one hand, and remodeled the gut microbiota dysbiosis accompanying calcium deficiency, on the other hand, with a significant decline in the abundance of pathogenic bacterium *Parasutterella*. Ultra-high-performance liquid chromatography–quadrupole-time of flight mass spectrometry (UHPLC-Q/TOF-MS) metabolomic analyses of cecum and humerus samples also suggested a normalization of isovaleric acid levels and holistic metabolic profiles. More interestingly, isoquercetin (IQ), a natural dietary flavonoid previously regarded as one of the desirable targets for hepatocellular carcinoma treatment (134), was innovatively validated by Wu M et al. (135) to have anti-osteoporosis properties in an OVX model. Long-term supplementation of IQ improved the disturbed gut microbiota and LPS-triggered inflammatory status, at the same time inhibiting the NF-κB signaling pathway, thereby alleviating or reversing bone loss outcome. In addition, *in vitro* experiments demonstrated that the effect of IQ was dose-dependent and achieved partly through the promotion of osteoblast proliferation. However, it is noteworthy that some substances listed above may be more than simply categorized as dietary supplements, which may also engage in more macroscopic modulation of dietary patterns.

#### 5.2.2 Dietary patterns

Osteoporosis and osteoarthritis are widely accepted as chronic diseases, making conventional therapies combined with lifestyle modifications indispensable. As an adaptive factor that is highly correlated with the gut–bone axis, elaborating the overall localized dietary patterns that affect bone health is critical. Dietary patterns focused on reducing inflammatory response *in vivo* through the intake of anti-inflammatory and antioxidative foods are thought to hold the potential of ameliorating osteoporosis (130). For example, the Mediterranean diet (MD), low glycemic index diet (LGID), and dietary approaches to stop hypertension (DASH) have been tested to diminish the levels of inflammatory factors such as C-reactive protein (CRP), IL-6, and TNF-α, but their detailed roles in the maintenance of bone quality have yet to be elucidated. The botanical constituent blackcurrant (BC) was noted to significantly and dose-dependently increase the abundance of *Ruminococcus* 2 in a clinical RCT that recruited 51 PMOP women with BC intake for six consecutive months (136). Further correlation analyses demonstrated the high correlation between *Ruminococcus* 2 and BMD maintenance in the high BC group, which highlighted *Ruminococcus* 2 as the key bacterium in bone protection. Also, the significant upregulation of plasma proteins (IGFBP4, fetuin-B, tetranectin, and vitamin K-dependent protein S) was tentatively proposed to be associated with osteoclast activity. He W et al. (137) administered milk calcium-fortified yogurt to OVX mice for 6 weeks, observed the modulation of the glycine pathway and the elevated *Lactobacilli*, and monitored the decrease in Clostridiaceae by metabolome and genome techniques, which were directly related to enhanced spinal BMD. The above results implied that yoghurt, a processed dairy product, may act as a calcium vector and benefit bone mineralization in PMOP. Liu Z et al. (47) revealed the harmful effects of chronic alcohol consumption on bones in that long-term intake of large amounts of ethanol affects the gut microbiota while stimulating the negative regulation of the gut–bone axis by 5-HT, which in turn induces the genesis of alcoholic osteoporosis in rats.

It is worth noting that prebiotics, in a broad sense, also belong to the category of dietary supplements while being incorporated into dietary patterns with relatively unclear boundaries. For instance, the fruit product dried prunes (138), conventionally categorized as a dietary supplement, has been shown to restore bone loss in OVX mice, as further studies have attributed this effect to polyphenols and carbohydrates, which are more explicitly identified as prebiotics. Furthermore, given the strong interaction of the prebiotic–gut microbiota axis, discussing prebiotics in the context of microbiota may make more sense, as described above. However, here, we give the concept of “Medicine Food Homology (MFH)” (139), which encompasses dietary-relevant structures that are postulated to have therapeutic effects in orthopedic diseases through the gut–bone axis. MFH is derived from the theory of traditional Chinese medicine (TCM), which reveals both the nutritional and medicinal value of many foods, and the preventive and therapeutic effects of these substances on orthopedic diseases corroborate this point. It is undeniable that MFH is currently regarded as a hotspot in food research and also a blue-ocean market with great potential. Nevertheless, whether food as medicine possesses real efficacy, and whether such efficacy can be presented in more quantitative and visualized indicators, is highly related to the subsequent clinical translation and industrial launch and is also the most worthwhile issue awaiting to be clarified in the field of bone health.

### 5.3 Drug application

The routine therapeutic drugs for osteoporosis include BPs, selective estrogen receptor modulators, and parathyroid hormone analogs, matched to the intervention of osteoporosis under different etiologies, fulfilling ideal efficacy while accompanied by certain adverse effects (33). Following the renaissance and promotion of TCM, mounting studies currently spotlight the seminal applications of herbs in the prevention and treatment of skeletal lesions and envisage their potential mechanisms *via* the gut–bone axis. Li K et al. (140) reviewed a variety of natural herbs such as *Achyranthes bidentata* Blume, *Ganoderma lucidum*, *Pueraria lobata*, and *Agaricus blazei* Murill, which were found to potentially affect Firmicutes and Bacteroidetes abundance, promote SCFA production, and modulate Treg/Th17 proportion, thereby indirectly working on bone maintenance. The function of puerarin, in particular, was also revealed by Li B et al. (141) through experimental methods such as μ-CT, 16S rRNA sequencing, and metabolomics. Results showed that puerarin restored the disturbed intestinal microecology by enriching amino acid metabolism, butyric acid generation, and LPS biosynthesis, herein alleviating osteoporosis. The above confirmed the clinical potential of puerarin as a phytoestrogen to remodel the bone microenvironment through the gut–bone axis and thus counteract PMOP. Korean red ginseng (KRG) and its processed products, senna granules (SG), have also been shown to prevent and alleviate osteoporosis *via* the gut–bone axis. Chargo NJ et al. (142) observed the effects of KRG extracts in preventing distal femur bone loss as well as significantly altering the composition of the gut microbiota in corticosterone-induced osteoporosis (GIO) mice. Kang HJ et al. (46) similarly observed a significant correlation between bacteria *Lactobacillus*, *rc4-4*, and *Alistipes finegoldii* and bone strengthening after the addition of KRG extracts in a mouse model of significant bone loss induced by intestinal dysbiosis following ABX, suggesting the involvement of the gut–bone axis in the role of KRG extracts in bone health. However, both studies appeared to find relatively limited effects of KRG extracts on modulating immune status and intestinal barrier function. In contrast, SG delivered better clinical benefits, which have been shown to be an efficient prescription for PMOP (42). SG intake not only systemically strengthened bone quality but also modulated the composition of the gut microbiota. At the cellular and molecular levels, the expression of femoral OPG and RANKL proteins, fecal SCFAs and colonic FOXP3 cells, and ZO-1 and Occludin proteins were elevated, together with the upregulation of serum cytokines IL-10 and TGF-β and the downregulation of IL-17 and TNF-α. The above results hinted that SG ameliorates osteoporosis *via* the gut–immune–bone axis as an ideal vehicle, which may account for its superiority over KRG extracts. Interestingly, some novel small molecules present in natural herbs have also been tentatively speculated to exert protective effects against osteoporosis through the gut–bone axis. For example, Icariin I (GH01) (143), extracted from the herb *Epimedium*, has been reported to significantly restore the intestinal barrier function, remodel the microbial composition, and regulate the host immune status in OVX mice, thereby effectively improving osteoporosis and bone loss, and has undergone a preliminary exploration in the innovative application of TCM.

## 6 Future prospects: innovating potential approaches for gut–bone axis to protect bone health

### 6.1 Disciplinary intersection with nano/molecular medicine

Based on the excellent biocompatibility, degradability, and mechanical properties of biomaterials, a variety of nanomaterials have been applied in fracture reposition, bone defect restoration, joint replacement, and bone tumor treatment. Nanomedicine, as a major branch of molecular medicine, has gradually become a novel therapeutic trend in the current treatment of orthopedic diseases by virtue of its high efficiency, precision, and low adverse effects (144). Zheng Y et al. (145) developed a propolis nanoemulsion (PNE), which can be administered to manage osteoporosis by means of targeted transgene modulation. Test results showed that the oral administration of PNE mediated a decrease in the abundance of *Streptococcus intestinalis* and an increase in the transgenic metabolite l-arginine, which further inhibited osteoblasts and enhanced osteoclasts through the gut–bone axis, exerting a significant anti-osteoporotic effect. Furthermore, Chen Y et al. (146) observed the anti-inflammatory and antibacterial activities of gold nanospheres (GNS), which were screened under scanning electron microscopy (SEM) for uniform particle distribution with a zeta-potential value of approximately −24.6 mV. The modulatory effect of GNS on intestinal homeostasis (Chao1, Shannon, and Simpson indices, PCoA, and F/B ratio) was demonstrated in an OVX mouse model of osteoporosis, which then restrained the metabolism of microbiota-related TMAO (betaine, choline, creatinine, carnitine, TMA, and TMAO) and the release of pro-inflammatory cytokines [TNF-α, IL-6, and granulocyte colony-stimulating factor (G-CSF)], thereby inhibiting bone loss induced by estrogen deficiency. Admittedly, the application of nanomedicine in osteoporosis shows definite potential in the context of medical–industrial integration. However, as an emerging discipline, the preparation and characterization, targeted delivery, and controlled release of nanomaterials remain a challenge, and the issues of biosafety, efficacy complexity, cost, and regulation also need to be optimized.

### 6.2 Engineering of extracellular vesicles

**Figure 4:**
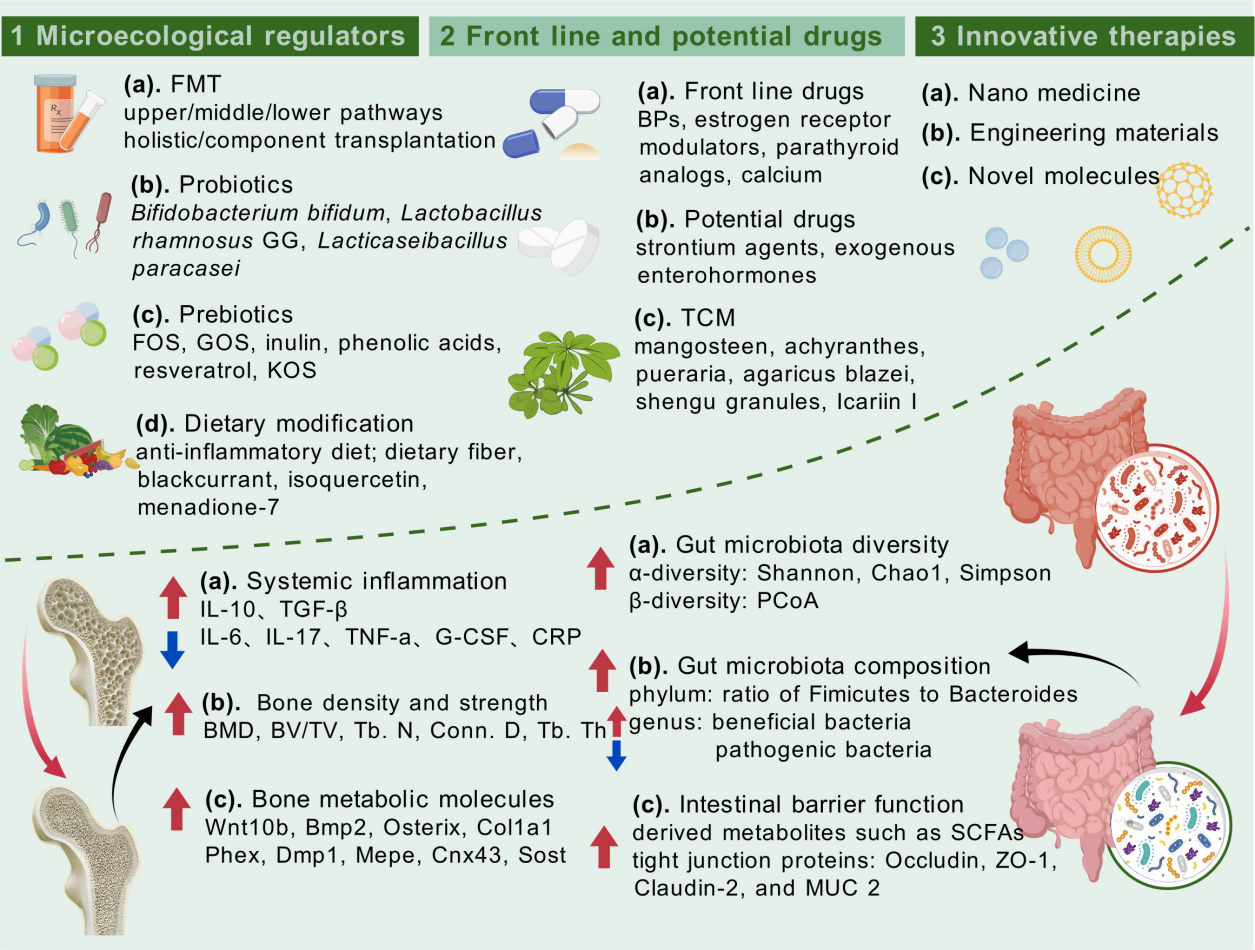
Intestinal microecology-targeted strategies for the management of osteoporosis and therapeutic effects on both bone health and intestinal homeostasis.

Extracellular vesicles (EVs) refer to vesicular structures with a membrane structure released by cells, including different subgroups such as exosomes, microvesicles, and apoptotic vesicles. These vesicles exist widely in blood, saliva, and urine, serving as an essential medium for intercellular communication (147). In recent years, the mechanism of EVs to prevent osteoporosis has been increasingly elucidated. It was reported that EVs from osteoblasts and MSCs are able to carry various signaling molecules, such as RNA, proteins, and lipids, thus regulating the balance of bone resorption/formation and promoting the reestablishment of bone tissue (148). For example, Lee KS et al. (149) extracted EVs from adipose tissue-derived stem cells (ASC-EVs), in which OPG, miR-21-5p, and let-7b-5p were highly enriched. Among them, OPG (decoy receptor for RANKL) blocked the interaction between RANKL (NF-κB ligand) and RANK, miR-21-5p downregulated Acvr2a, and let-7b-5p significantly downregulated the expression of osteoclast-related genes, which ultimately inhibited the germination and differentiation of osteoclasts. In addition to *in situ* effects on bone tissue, some natural or engineered EVs also participated in gut microecology in the anti-osteoporotic process, suggesting the involvement of the gut–bone axis. Hao H et al. (150) explored the effects of milk-derived EVs (mEVs) on osteoporosis in OVX mice and observed that the supplementation of mEVs promoted the colonization of anabolic *Bacillus* spp. associated with SCFAs; upregulated the levels of intestinal acetic, propionic, and valeric acids; and inhibited the expression of serum TNF-α and IL-17 while downregulating the factors related to osteoclast differentiation in bone tissue to alleviate osteoporosis symptoms. More excitingly, Liu H et al. (151) used recombinant *Escherichia coli* Nissle 1917 to construct a fusion overexpression system of BMP-2 and CXCR4 with ClyA, a kind of bacterial EV (BEV) surface protein, and finally designed the engineered BEVs-BMP-2-CXCR4. BEVs obtained through this synthetic biology technique significantly promoted the differentiation of bone marrow MSCs toward osteoblasts and effectively prevented osteoporosis in OVX mice. In general, EVs exhibit superior bone-targeting and drug-loading capabilities as a novel cell-free therapeutic, which will play a greater role in bone health with updated extraction and purification processes (**Figure 4**).

### 6.3 Future technologies oriented by gut–bone axis

Beyond referring to the past and taking stock of the present to achieve anti-osteoporosis effects through the gut–bone axis, it is equally of great significance to optimize tools for future technologies and to address the issues that exist along the way. Actually, studies have already been conducted to prospectively focus on bone loss in future survival environments, and Bedree JK et al. (152) examined the correlation between bone loss during spaceflight and gut microbiome, as well as specific host metabolites. Contextualizing the decline in BMD and disturbances in bone homeostasis according to Rodent Research 5 implemented by National Aeronautics and Space Administration (NASA) in the Deep Space Travel Experiment (DSTE), the study employed whole-genome sequencing, 16S rRNA analysis, and liquid chromatography–tandem mass spectrometry (LC-MS) metabolomics to demonstrate the relative abundance alterations of bacterial strains (*Lactobacillus murinus* and *Dorea* sp.) and the functional gene cluster enrichment of metabolites (lactic acid, leucine/isoleucine, and glutathione) when exposed to microgravity. The exploration of spaceflight-related bone loss at the present time represents a more cutting-edge vision, but shortcomings also exist. Except for the possible lack of sophistication in subject selection, the detection tools adopted and the potential mechanisms explored remain largely within the standard paradigm. While the latter appears to be a common problem in the research of the gut–bone axis affecting osteoporosis at the current level, the limitations are not yet evident.

As summarized previously, a number of studies have revealed the impact of the gut microbiota on bone health and highlighted, for example, the role of bone metabolism by modulating the immune system, metabolic pathways, and hormonal dynamics. However, the specific and comprehensive molecular mechanisms, or whether there are associations and crosstalk between these different pathways, remain unclarified. With the advancement of molecular biology and genetics, networking the biological information on the effects of gut microbes on bone metabolism is possible, which will also provide a theoretical basis for the formulation of novel therapies and drugs. In addition, consistent with the current state of osteoporosis worldwide, mounting relevant basic and clinical trials are underway. The former centers on animal models, which illustrate valuable insights into the potential mechanisms by which the gut–bone axis influences osteoporosis, but more evidence is required to translate the findings into human clinical practice. However, due to the complexity and ethical constraints of clinical trials, it is difficult for the latter to conduct large-scale and long-term follow-up, and in contrast, most clinical RCTs aim at the validation of symptomatic drugs such as calcium, vitamin D, and BPs, while the microecological modifiers that target the gut–bone axis are seldom screened. More high-quality, large-scale, and multi-centered clinical trials to prove the efficacy and safety of gut microbiota modulation in the treatment of orthopedic diseases are necessary.

The next concern is methodology. Current gut microbiota monitoring relies primarily on 16S rRNA sequencing, with metabolomics or proteomics being additionally supplemented in some studies. These tools collect microbial information on certain facets at the genetic and transcriptional levels, but suffer from limited efficiency and low visualization levels and at the same time fail to achieve a dynamic description of the spatiotemporal distribution of the gut microbiota. As a result, there is still uncertainty as to whether the assessment of the intestinal microenvironment can accurately reflect bone health. The development of more dynamic, objective, and visible assessment methods and storage tools is warranted to review gut microbiota alterations and their impact on bone health *via* the gut–bone axis and to facilitate clinical decision-making by delivering more detailed interactive information. Otherwise, given the individualized differences in gut microbiota composition and bone quality, the population’s responses to the same therapy can be variable. Herein, a feasible strategy would be to investigate the possibility of categorizing osteoporosis populations according to the gut microbiota, a more explicit and non-invasive characterization, for pre-osteoporosis prophylaxis and more precise cause-specific and symptomatic treatment based on subgroup characteristics. Individualized interventions based on the gut microbiota may enhance therapeutic efficacy while minimizing side effects, which would benefit the majority of middle and older-aged adults, as well as some young children with skeletal disorders. Of course, all of the above cannot be achieved without interdisciplinary collaboration between orthopedics and a variety of fields, including microbiology, genetics, immunology, and endocrinology, which will help comprehend the relationship between the gut microbiota and osteoporosis and expand the therapeutic spectrum of orthopedic diseases.

## 7 Conclusion

**Figure 5:**
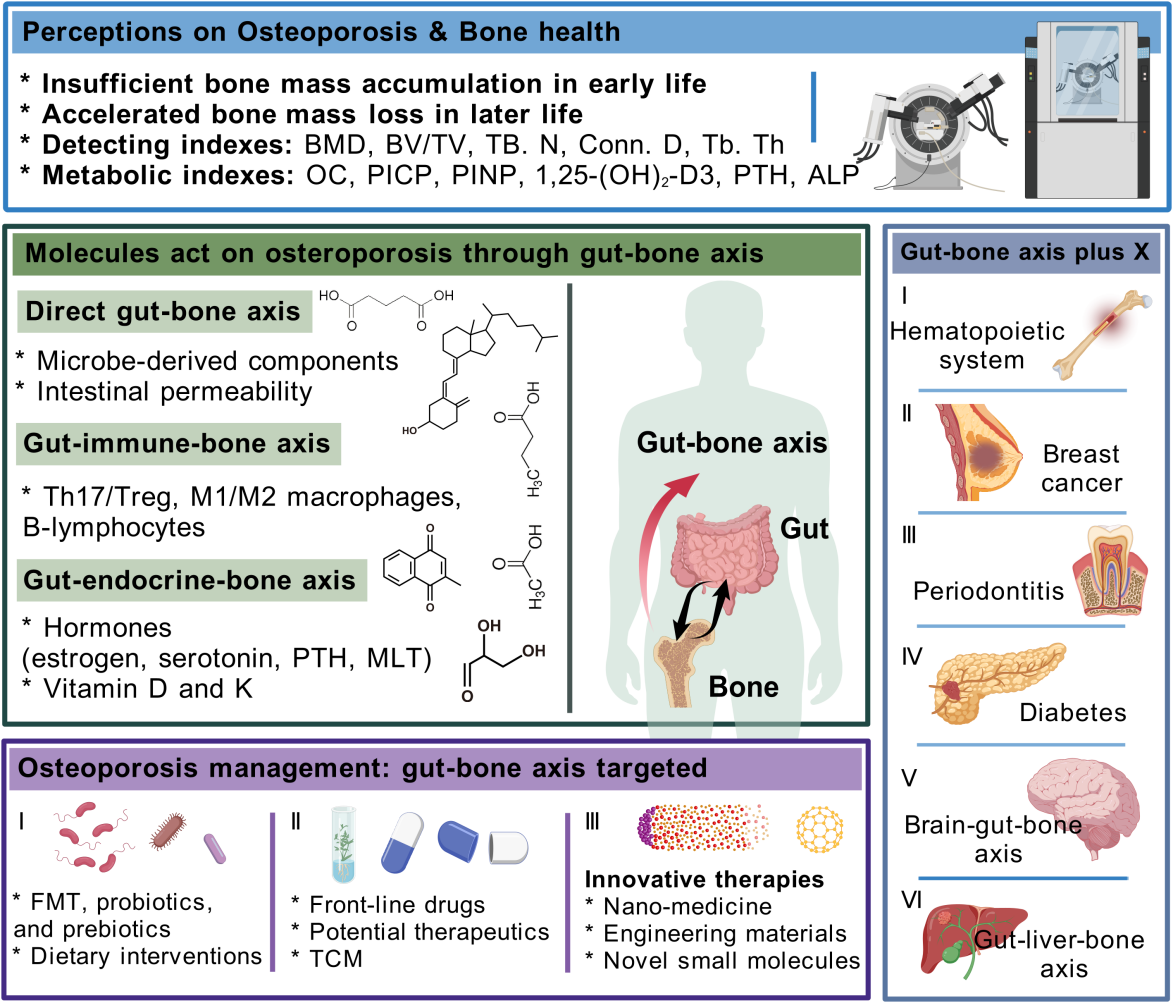
Integral elaboration on appraisal of osteoporosis, how gut–bone axis influences bone health and interacts with other organs, and potential interventions for osteoporosis that target the gut–bone axis.

In conclusion, this review focused on osteoporosis, the most common bone disease jeopardizing bone health, and described its causative factors, mechanisms, and diagnostic and therapeutic tools and their existing shortcomings. Clinical evidence on the involvement of gut-related factors in osteoporosis led us to undertake the core concept of the gut–bone axis and systematically summarize that the intestinal intrinsic structure, gut microbiota, and derived metabolites, with the immune and endocrine routes as the intermediate transitions, attain a bidirectional interplay with skeletal tissue through the gut–bone axis. Based on the holistic structure and function of the organism, we further propose the framework of the gut–bone axis plus X, which provides some reference for the derivation and qualification of the gut–bone axis, helps to clinically interpret the gut–bone axis stimulated by organs such as the brain and liver, and precisely facilitates the treatment of differentiated individuals with osteoporosis. Furthermore, this review details FMT, probiotics and prebiotics, and dietary patterns as intervention strategies targeting the gut on osteoporosis; summarizes conventional medications, potential therapeutic drugs, and TCM that exert anti-osteoporosis effects *via* the gut–bone axis; and illustrates the superiority of nanomedicine, engineered biomaterials, and future technologies in the promotion of bone health given the interdisciplinary strengths of orthopedics. The above portrayal of the gut–bone axis in anti-osteoporosis, on the one hand, stimulates a broader management mindset for active health of the skeleton, and, on the other hand, highlights the key involvement of intestinal factors in systemic pathogenesis, which is expected to provide theoretical basis for disease intervention strategies that target the gut and the clinical implementation of micro-ecological preparations (**Figure 5**).
